# The Inhibitory Effect of Phycocyanin Peptide on Pulmonary Fibrosis *In Vitro*

**DOI:** 10.3390/md20110696

**Published:** 2022-11-06

**Authors:** Run-Ze Liu, Wen-Jun Li, Juan-Juan Zhang, Zheng-Yi Liu, Ya Li, Chao Liu, Song Qin

**Affiliations:** 1 Yantai Institute of Coastal Zone Research, Chinese Academy of Sciences, Yantai 264003, China; 2 Center for Ocean Mega-Science, Chinese Academy of Sciences, Qingdao 266071, China; 3 Yantai Jiahui Biotech Co., Ltd., Yantai 264003, China

**Keywords:** phycocyanin, phycocyanin peptide, pulmonary fibrosis, inflammatory, oxidative stress, heme oxygenases-1

## Abstract

Phycocyanin is an excellent antioxidant with anti-inflammatory effects on which recent studies are growing; however, its specific target remains unclear. Linear tetrapyrrole compounds such as bilirubin have been shown to lead to the induction of heme oxygenase 1 expression *in vivo*, thus achieving antioxidant and anti-inflammatory effects. Phycocyanin is bound internally with linear tetrapyrrole phycocyanobilin in a similar structure to bilirubin. We speculate that there is probably a way of inducing the expression of heme oxygenase 1, with which tissue oxidative stress and inflammation can be inhibited, thus inhibiting pulmonary fibrosis caused by oxidative damage and inflammation of lung. By optimizing the enzymatic hydrolysis process, phycocyanobilin-bound phycocyanin peptide were obtained, and its *in vitro* antioxidant, anti-inflammatory, and anti-pulmonary fibrosis activities were investigated. The results show that the phycocyanobilin peptide was able to alleviate oxidative and inflammatory damage in cells through the Keap1-Nrf2-HO-1 pathway, which in turn relieved pulmonary fibrosis symptoms.

## 1. Introduction

Idiopathic pulmonary fibrosis (IPF) is a progressive form of pulmonary fibrosis in which the structure of the alveoli is damaged and gas exchange in the lungs is impaired, eventually leading to respiratory failure and death [1]. At present, the etiology and specific pathogenesis of IPF remains poorly understood. The current mainstream view is that IPF results from alveolar epithelial cell damage and consists of oxidative stress damage [2] and inflammatory damage by various interactive genetic and environmental factors. An epithelial cell injury would trigger abnormal communication between epithelial cells and fibroblasts, induce activation of myofibroblasts, secrete a large amount of extracellular matrix (ECM), deposit in the lungs, and consequently bring about non-structural remodeling of the lungs and fibrosis [3]. Unfortunately, there is no practically effective treatment for IPF. Conventional anti-pneumonia drugs, such as prednisone, dexamethasone, and other corticosteroids have no obvious effect on pulmonary fibrosis but serious side effects [4]. In recent years, new drugs such as nintedanib and pirfenidone can alleviate the symptoms of pulmonary fibrosis to a certain extent, but serious side effects (nausea, abdominal pain, diarrhea, photosensitivity, and nervous system abnormalities) are unavoidable, thus hampering the clinical application [5,6]. Therefore, looking for a safe, less toxic, and side-effect-free drug for the treatment of IPF has become an urgent task for drug research institutions.

Inflammation is widespread in the organism of IPF patients, which is the host’s defense action against injury, oxidative damage, or infectious pathogens, as well as adaptive immune response [7]. In the innate immune response, macrophages play a key role as an immune cell. By secreting a variety of inflammatory factors, including tumour necrosis factor (TNF) and interleukins (IL), macrophages are able to clear pathogens from the body [8,9]. Moderate amounts of inflammatory factors can participate in the body’s immune response, while excessive production of inflammatory factors can damage the body and lead to various inflammatory diseases such as sepsis, pulmonary fibrosis, diabetes, and atherosclerosis [10,11]. Therefore, regulating the activity of secreting inflammatory factors in macrophages can inhibit pulmonary fibrosis caused by tissue inflammation to a certain extent.

Heme oxygenase 1 (HO-1) is an antioxidant-induced enzyme responsible for the breakdown of heme into biliverdin [12]. Earlier studies have identified HO-1 as having anti-inflammatory effects and the ability to modulate various immune cells, including macrophages [13,14,15], making HO-1 an important target in the treatment of inflammation and even pulmonary fibrosis induced by inflammation. Being similar in linear tetrapyrrole structure to that of bilirubin, phycocyanobilin (PCB) can induce the expression of HO-1. However, current bilirubin analogues are likely to cause jaundice during clinical administration, and have disadvantages such as toxic side effects and low bioavailability, which has limited the application of HO-1 targeting in anti-inflammatory and anti-fibrosis treatment.

**Figure 1:**
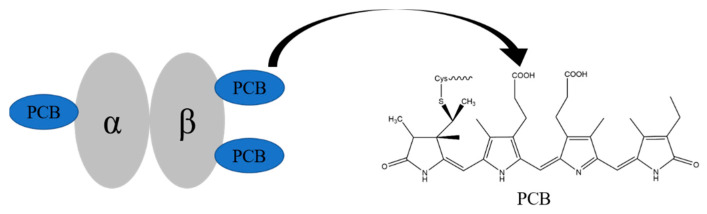
The structures of phycocyanin monomer and phycocyanobilin.

Phycocyanin (PC) is a light-harvesting protein widely found in algae, which is soluble in water. It is composed of linear tetrapyrrole pigment PCB through thioether bond with apoprotein α and β. In PC, three PCBs are respectively connected to Cys-84 of the α chain, Cys-84, and 155 of the β chain [16] (Figure 1) and are wrapped in the hydrophobic core inside.

PC has many biological activities as an antioxidant, anti-inflammatory, and anti-pulmonary fibrosis agent [17,18]. Li et al. [18] used PC to treat mice that had bleomycin-induced pulmonary fibrosis, and found that PC could pass through the TLR2-MyD88-NF-κB signaling pathway to effectively inhibit the bleomycin-induced pulmonary fibrosis. In addition, Liu et al. [19] used PC to cure mice that had radiation-induced acute liver injury, and they found that PC significantly up-regulated the expression of Nrf2 and the downstream gene HO-1, which reduced the liver injury. Therefore, we believe that the activity of PC is in fact from PCB that is contained within PC. PCB in PC would be exposed and released after gastrointestinal digestion and be in full contact with the environment. As it features a linear tetrapyrrole structure, the active groups are the same as bilirubin to induce the expression of HO-1 against oxidation, inflammation, and pulmonary fibrosis [20,21].

PC has the advantages of non-toxicity and good water solubility, and it contains PCB, which is a satisfactory material for preparing the HO-1 inducer. Therefore, we enzymatically hydrolyzed PC and decomposed it into small peptides in 10 amino acids, by which PCB that was fully exposed and phycocyanin peptide connected with PCB was formulated. In addition, the antioxidant activity, LPS-induced RAW264.7 macrophage inflammation, and TGF-β1-induced pulmonary fibrosis inhibition were determined by *in vitro* experiments.

## 2. Results

### 2.1. Enzymatic Hydrolysis of Phycocyanin and Its Absorption Spectrum and Molecular Weight Determination

PCB in PC is very sensitive when ambient pH, temperature, etc., change and the color during enzymatic hydrolysis could easily vary, thus affecting the final product. To avoid the color change after enzymatic hydrolysis and ensure the activity of phycocyanin peptide, after a large number of screening tests, we applied the composite enzyme MC101 to the enzymatic hydrolysis process of PC for the first time. The activity of MC101 was high, and the yield rate of phycocyanin peptide reached 96% in blue color. The color of the phycocyanin peptide after enzymatic hydrolysis was lighter than that of PC, showing light blue. We think that phycocyanin peptide contained a peptide fragment bound to PCB. Apparently, the PC solution was purplish blue and the solution of phycocyanin peptide after enzymatic hydrolysis turned blue. This is because phycocyanin has fluorescence characteristics, so its solution presents a purple color due to the red and blue mix. The active structure of the phycocyanin is destroyed after enzymatic hydrolysis, so the fluorescence disappears, leaving only the blue color of PCB. PCB is a fat-soluble substance and does not dissolve in water when it exists alone. However, the phycocyanin peptide after MC101 complex enzymatic hydrolysis had good water solubility, and could be quickly dissolved even at high concentrations, which broadened the application range of the phycocyanin peptide.

**Figure 2:**
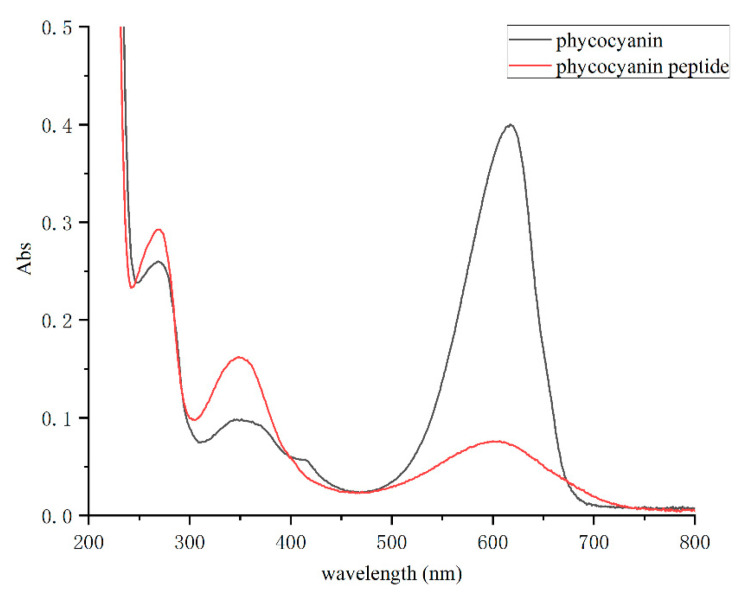
The absorption spectra of phycocyanin and phycocyanin peptide.

By configuring the *Spirulina* PC and phycocyanin peptide into a same-concentration solution and scanning in the full wavelength range of 200–800 nm, the absorption spectrum of the phycocyanin peptide was obtained (Figure 2). The absorption peaks of PC distributed mostly at 620 nm (chromophore absorption) and 280 nm (aromatic amino acid absorption), and another peak at 360 nm reflects the PCB conjugate. In the full-wavelength scan, it was shown that the absorbance peak of phycocyanin peptide at 620 nm decreased obviously, while those at 280 nm and 360 nm increased. The properties of the absorption spectrum are very similar to that of the PCB standard. Therefore, it shows that the PC had been fully decomposed into phycocyanin peptides, and the enzymatic hydrolysis was sufficient.

Molecular weight distribution of the phycocyanin peptide is shown in Table 1. The average molecular weight of the phycocyanin peptide is 583 Da, which is much smaller than 40 kDa of PC, while the calculated value from average molecular weight of amino acids in the protein was 110 Da. Therefore, we believe that the obtained enzymatic peptide contained 5–6 amino acids.

Subsequently, the phycocyanin peptide was analyzed by HPLC-MS in order to obtain information on the fragments of peptides bound with PCB. The HPLC-MS results are shown in the supplemental Table S1. From the results, it can be seen that PC, after enzymatic digestion, had 36 fragments containing PCB of different lengths, of which 9 were from the PC α subunit with lengths ranging from 9–16 amino acids and 27 were from the PC β subunit with lengths ranging from 3–21 amino acids. Considering that fragments with many amino acids would not be easily absorbed, 11 fragments with amino acid numbers less than 10 were obtained after further screening (Table 2), of which 1 was from the PC α subunit and 10 from the PC β subunit. By comparing the obtained sequences, it was shown that the PCB of the fragment from the α subunit binds to Cys84. Among the 10 fragments from the β subunit, 5 PCB bound to Cys82 and 5 PCB to Cys153 (Table 2).

### 2.2. Antioxidant Activity of Phycocyanin Peptide

PC has been shown to have excellent antioxidant activity [17], but whether the enzymatic-hydrolyzed phycocyanin peptides have similar or better activity is currently unknown. Therefore, we compared the *in vitro* antioxidant activities of PC and phycocyanin peptides to understand in-depth the activity of the phycocyanin peptide.

#### 2.2.1. Superoxide Anion Scavenging Rate

**Figure 3:**
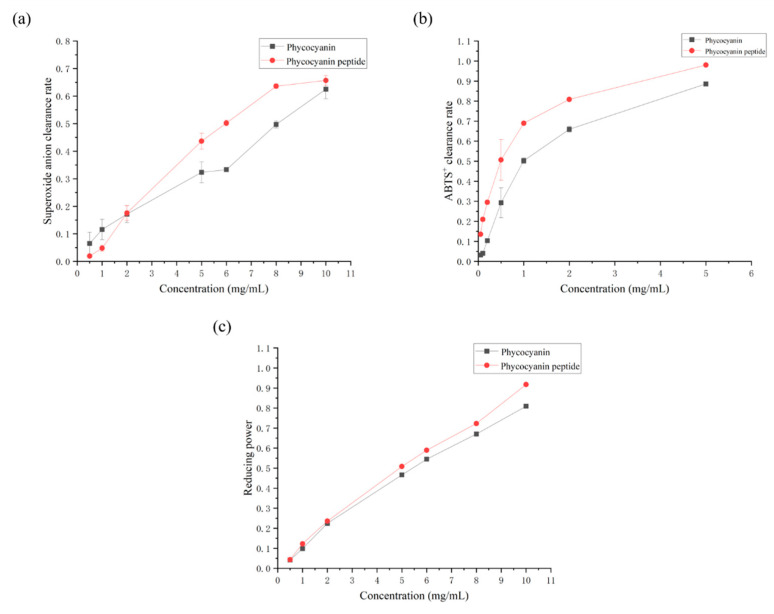
Antioxidant activity of phycocyanin peptide: (**a**) superoxide anion clearance; (**b**) ABTS^+^ clearance; (**c**) total reducing power.

Superoxide anion ($O_{2}^{-}$) is a kind of reactive oxygen species (ROS) produced in organisms through respiration and other pathways [22,23]. Excessive $O_{2}^{-}$ can cause damage to the body, produce oxidative stress, and trigger various pathological processes, such as protein denaturation, nucleotide damage, etc. [24,25]. Figure 3a shows that the $O_{2}^{-}$ scavenging ability of PC or the phycocyanin peptide enhanced as their respective concentrations increased. Overall, the $O_{2}^{-}$ clearance rate of the phycocyanin peptide is greater than that of that of PC. The curves were linearly fitted, and the IC50 values of PC and the phycocyanin peptide were 8.15 mg/mL and 6.65 mg/mL, respectively. The $O_{2}^{-}$ clearance rate of the phycocyanin peptide after enzymatic hydrolysis was greatly improved from the clearance rate of PC.

#### 2.2.2. ABTS^+^ Scavenging Rate

Reduced ABTS is a colorless substance that can be oxidized to blue-green ABTS^+^ free radicals by oxidants. ABTS^+^ free radicals are very stable and have a maximum absorption peak at 734 nm. Antioxidants that are able to donate hydrogen can react with ABTS^+^ and can turn them back into colorless ABTS. Therefore, ABTS is often used as an *in vitro* antioxidant index to judge the antioxidant capacity of a sample [26].

The scavenging ability of PC or the phycocyanin peptide of ABTS^+^ is shown in Figure 3b. It was observed that PC and the phycocyanin peptide had different degrees of scavenging effect on ABTS^+^. In the selected concentration range, with the increase of concentration, the clearance of PC and the phycocyanin peptide of ABTS^+^ was logarithmically correlated. At 5 mg/mL, the scavenging rates of PC and the phycocyanin peptide of ABTS^+^ were 88.58% and 98.02%, respectively. The IC_50_ values of PC and the phycocyanin peptide were 0.96 and 0.42 mg/mL, respectively.

#### 2.2.3. Total Reducing Power

The reducing power refers to the color oxidation reaction that determines whether the sample can become a good electron donor. The oxidation resistance of a substance depends on the reducing power, so the oxidation resistance can be judged by measuring the reducing power of the sample. Potassium ferricyanide is an oxidizing complex. After reacting with reducing substances, it produces colorless potassium ferrocyanide. Potassium ferricyanide reacts with FeCl_3_ to produce Prussian blue. There is a characteristic absorption peak at 700 nm. Therefore, the relative reduction power of the sample can be ascertained by an absorbance measurement at 700 nm [27].

The total reducing power of PC or the phycocyanin peptide is shown in Figure 3c. In the range of 0.5–10 mg/mL, the reducing power of PC or the phycocyanin peptide increased with the increase of its own concentration, and the reducing power was positively correlated to the concentration. The reducing power of PC and the phycocyanin peptide was 0.806 and 0.914 at a sample concentration of 10 mg/mL, respectively. In addition, the difference in total reducing power between PC and the phycocyanin peptide was not obvious at low concentrations (Figure 3c). As the concentration increased, the phycocyanin peptide enzymatically hydrolyzed with MC101 showed better reduction than that of PC.

### 2.3. Anti-Inflammatory Activity of Phycocyanin Peptide

#### 2.3.1. Effect of Phycocyanin Peptide on Survival Rate of RAW264.7 Cells

**Figure 4:**
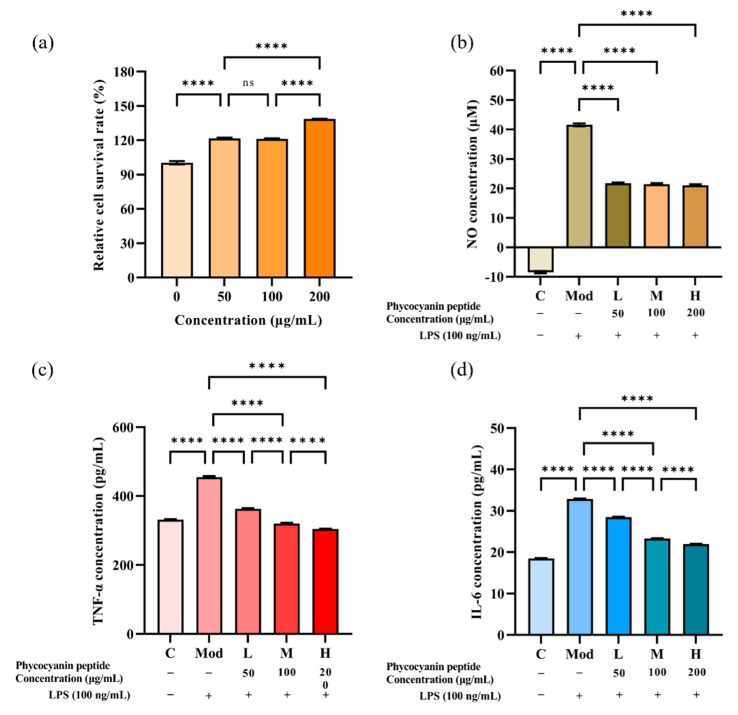
Anti-inflammatory activity of phycocyanin peptide: (**a**) relative cell survival rate of RAW264.7 macrophages; (**b**) NO scavenging ability; (**c**) TNF-α scavenging ability; (**d**) IL-6 scavenging ability. C: Control; Mod: Model; L: Low-dose (50 μg/mL); M: Medium-dose (100 μg/mL); H: High dose (200 μg/mL). *n* = 3, mean ± SD. **** *p* < 0.0001; ns *p* > 0.05.

The CCK-8 assay was applied to exclude drug interference with cell status and to provide a basis for subsequent selection of the appropriate concentration. Taking the relative cell viability ≥90% as the criterion of no cytotoxicity, the phycocyanin peptide did not inhibit the growth of macrophages (Figure 4a). Overall, the phycocyanin peptide slightly promoted the proliferation of RAW264.7. The relative survival of cells at 200 μg/mL of phycocyanin peptide was approximately 138%, which proved that the phycocyanin peptide had no obvious cytotoxicity and was safe.

#### 2.3.2. Inhibitory Effect of Phycocyanin Peptide on NO

NO is a type of cytokine which can mediate many biological functions. Macrophage-derived NO plays a major role in physiology and pathology. A proper amount of NO can promote the immune response of the body, while the over expression of NO could cause inflammation such as rheumatoid arthritis, atherosclerosis, tissue damage, etc. [28]. Therefore, the expression level of NO determines the induction or inhibitory effect of phycocyanin peptides on inflammation.

To investigate the inhibitory effect of phycocyanin peptides on NO, the effect of phycocyanin peptides on NO release from LPS-induced RAW264.7 cells was examined using the Griess method. Depending on the results of the CCK-8 experiment, the concentration of phycocyanin peptides was set at 50, 100, and 200 μg/mL. As shown in Figure 4b, the release of NO in the supernatant of RAW264.7 macrophages after LPS induction was increased dramatically compared with the control group, reaching 41.58 μM. After the intervention of phycocyanin peptides, the expression level of NO in the supernatant of RAW264.7 cells was effectively inhibited. As the concentration increased, the inhibitory effect of phycocyanin peptides on NO showed an upward trend. At a concentration of 50 μg/mL, the NO concentration was 21.75 μM, and the inhibitory rate of phycocyanin peptides on NO was 39.63%. After the concentration was raised to 200 μg/mL, the NO concentration was 20.98 μM, and the NO inhibition rate was 41.16%.

#### 2.3.3. Inhibitory Effect of Phycocyanin Peptide on TNF-α and IL-6

As inflammatory factors secreted by macrophages, TNF-α and IL-6 overexpression often cause the development of many diseases [29,30]. Therefore, by detecting changes in the content of TNF-α or IL-6, the anti-inflammatory ability of phycocyanin peptides can be determined.

Based on the NO inhibition test, we examined the effect of phycocyanin peptides on the levels of IL-6 and TNF-α expression in the cell supernatant using the Elisa method and further validated their anti-inflammatory activity. The results are presented in Figure 4c,d. In terms of TNF-α, the induction of LPS promoted its expression, being increased from 331.29 pg/mL in the control to 454.54 pg/mL. After treatment with phycocyanin peptides, the expression of TNF-α was inhibited. Compared with the model group, which was added with 100 ng/mL LPS, the inhibitory effect of phycocyanin peptides on TNF-α was enhanced with the increase of the concentration. At 50 μg/mL, the TNF-α decreased to 362.94 pg/mL, and the relative inhibition rate was 74.32%. At 200 μg/mL, TNF-α returned to the level of the control group, or even slightly lower (304.68 pg/mL), with a relative inhibition rate of 100%. The effect of phycocyanin peptides on IL-6 expression in macrophages was similar to that of TNF-α, and the inhibitory effect continued to increase with the concentration increase. The concentration of IL-6 in the 50 μg/mL low-dose group decreased from 32.87 to 28.49 pg/mL, and the relative inhibition rate was 30.44%. The inhibitory effect was additionally enhanced at a high concentration of 200 μg/mL; after that, the IL-6 concentration was reduced to 21.95 pg/mL and the relative inhibition rate was 75.76%. The results show that phycocyanin peptides effectively suppressed the TNF-α and IL-6 expressions, and the suppression effect was gradually enhanced with increasing concentration. At a high concentration of 200 μg/mL, phycocyanin peptides restored TNF-α to normal levels. Although the recovery effect of IL-6 was not as great as that of TNF-α, the inhibition rate of IL-6 reached 75.76%, which proved that the obtained phycocyanin peptides played an anti-inflammatory role *in vitro* and had a good effect.

### 2.4. Anti-Pulmonary Fibrosis Activity of Phycocyanin Peptide

#### 2.4.1. A549 Cell Morphology and Collagen I Expression

**Figure 5:**
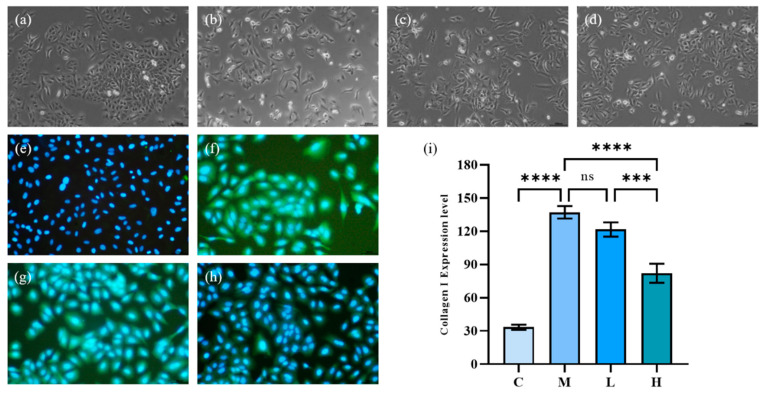
The effect of phycocyanin peptides on the morphology and Collagen I expression of A549 cells induced by TGF-β1: (**a**) cell morphology of control group; (**b**) cell morphology of model group; (**c**) cell morphology of low-dose group; (**d**) cell morphology of high-dose group; (**e**) expression of Collagen I in control group; (**f**) Collagen I of model group expression; (**g**) Collagen I expression in the low-dose group (10 μg/mL); (**h**) Collagen I expression in the high-dose group (30 μg/mL); (**i**) Collagen I expression level. C: the control group; M: the model group; L: the low-dose group (10 μg/mL); H: the high dose group (30 μg/mL). *n* = 3, mean ± SD. *** *p* < 0.001; **** *p* < 0.0001; ns *p* > 0.05.

Under the induction of TGF-β1, human lung epithelial cells (A549) can be polarized. The cells present a relatively slender spindle in shape, and the epithelial-mesenchymal transition (EMT) would occur, showing fibrotic activity. As Figure 5a–d show, the morphology of A549 cells after TGF-β1 significantly changed from original cobblestone-like cells to obvious spindle-shaped cells, and the cells were elongated. After treatment with phycocyanin peptides, the morphological recovery of A549 cells at low doses was not obvious, and most of the cells remained in spindle shape, while at high doses, the morphological recovery of A549 cells was more obvious, being similar to the control group.

A very significant symptom of IPF is the excessive deposition of extracellular matrix between tissues. Excessive deposition of ECM could aggravate the development of IPF. Collagen I, as the main component of ECM, plays a critical role in the excessive deposition of ECM. Collagen is not easy to be degraded as its conversion rate is very slow and has strong resistance to common proteases. Therefore, once collagen is produced, it is difficult to be removed from the human body, thereby continuously aggravating IPF. Therefore, Collagen I is an important indicator of IPF.

The effect of phycocyanin peptides is shown in Figure 5e–h in which the blue part was the nucleus of A549 cells, and the green, fluorescent part was Collagen I. After 72 h of TGF-β1 induction, the Collagen I expression of A549 cells increased significantly. Low-dose phycocyanin peptide had a certain alleviating effect on Collagen I expression, but the effect was not obvious. At high doses, phycocyanin peptides presented a better inhibitory effect on Collagen I expression, while the green fluorescence faded in general.

#### 2.4.2. Nrf2, NQO1 and HO-1 Expression

It has been proven that HO-1 has excellent antioxidant and anti-inflammatory activities and can modulate the activity of various immune cells. The induction of HO-1 is inseparable from the metabolism of bilirubin. Nam et al. demonstrated that bilirubin could induce the expression of HO-1 through the Keap1-Nrf2 pathway [21]. PCB in phycocyanin peptide is very similar in structure to bilirubin, so we therefore hypothesize that the anti-inflammatory activity of the peptides derives from the modulation of HO-1.

NAD(P)H: quinone oxidoreductase 1 (NQO1) is also an essential factor in the Keap1-Nrf2 signaling pathway. Through the reduction reaction, NQO1 can catalyze the reduction of quinones into hydroquinones and promote the metabolism of quinones, thereby avoiding the production of ROS by semihydroquinones and playing an antioxidant role in cells [31].

**Figure 6:**
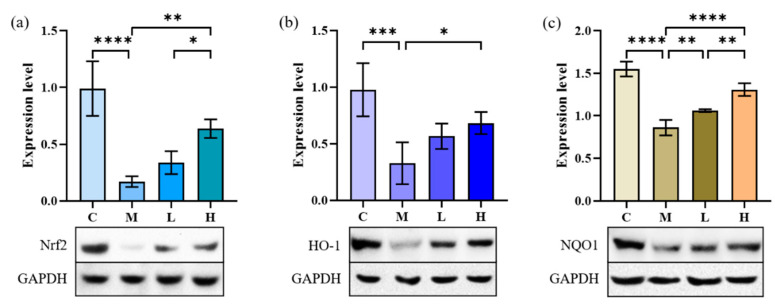
The effect of phycocyanin peptides on the expression of Nrf2, HO-1, and NQO1 in A549 cells induced by TGF-β1: (**a**) Nrf2 expression; (**b**) HO-1 expression; (**c**) NQO1 expression. C: the control group; M: the model group; L: the low-dose group (10 μg/mL); H: the high dose group (30 μg/mL). n = 3, mean ± SD. * *p* < 0.05; ** *p* < 0.01; *** *p* < 0.001; **** *p* < 0.0001.

After induction by TGF-β1, the levels of NQO1, Nrf2, and HO-1 in A549 cells were significantly decreased (Figure 6, *p* < 0.001), and the cells produced an inflammatory response. After treatment with 10 and 30 μg/mL of phycocyanin peptides, the three indexes all increased to different degrees, and they all recovered to more than 60% of the control group. Statistical analysis showed that the recovery of Nrf2, NQO1, and HO-1 was related to the intervention of phycocyanin peptides (*p* < 0.05); however, with the exception of NQO1, the increase of Nrf2 and HO-1 did not appear to be related to the concentration of phycocyanin peptides (*p* > 0.05).

#### 2.4.3. EMT-Related Proteins Expression

EMT refers to the process of epithelial cells that are transformed into mesenchyme cells and plays a critical role in tissue inflammation and fibrosis. TGF-β1 can induce the EMT process of A549 cells through the NF-κB pathway by expressing landmark proteins such as α-SMA, vimentin, N-cadherin, and E-cadherin. The effect of phycocyanin peptides on pulmonary fibrosis can be studied by measuring the expression of several marker proteins in the cell supernatant. The expressions of α-SMA, N-cadherin, and vimentin can advance the development of IPF during the occurrence of pulmonary fibrosis. E-cadherin can maintain the connection among epithelial cells. When E-cadherin levels are down-regulated, the connection among epithelial cells would be destroyed and the adhesion lost, which would greatly increase the migration ability of cells and changes their morphology, eventually leading to the occurrence of fibrosis.

**Figure 7:**
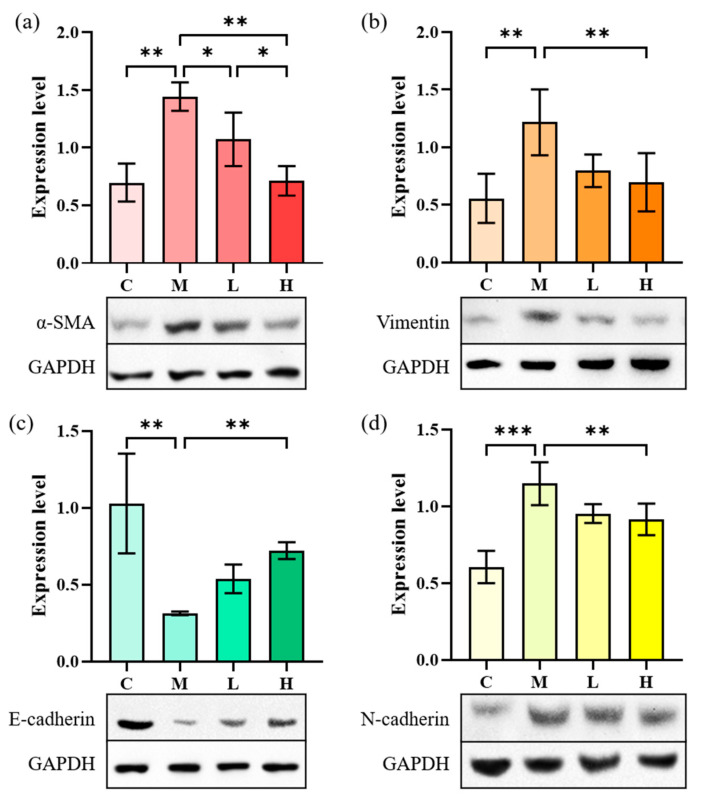
The effect of phycocyanin peptides on the expression of EMT-related proteins in A549 cells induced by TGF-β1: (**a**) α-SMA expression; (**b**) Vimentin expression; (**c**) N-cadherin expression; (**d**) E-cadherin expression. C: the control group; M: the model group; L: the low-dose group (10 μg/mL); H: the high dose group (30 μg/mL). n = 3, mean ± SD. * *p* < 0.05; ** *p* < 0.01; *** *p* < 0.001.

Figure 7 shows that the α-SMA content in A549 cells after induction of TGF-β1 increased from 0.31 to 0.88, that of N-cadherin increased from 0.43 to 0.86, and the expression of vimentin increased from 0.41 to 0.89. Meanwhile, the expression of E-cadherin decreased from 1.08 to 0.58, indicating that the EMT process occurred in A549 cells. After treatment with low-dose phycocyanin peptide, the expressions of α-SMA (0.80) and N-cadherin (0.85) were slightly down-regulated, that of E-cadherin (0.80) was significantly recovered, while that of vimentin (1.02) slightly increased. The expressions of α-SMA (0.61), N-cadherin (0.59), and vimentin (0.77) at high concentrations decreased largely, while that of E-cadherin (0.92) further increased, indicating that phycocyanin peptides could affect cell EMT *in vitro* with a significant reversal effect.

#### 2.4.4. HFL-1 Cell α-SMA Expression

During the development of IPF, fibroblasts can be transformed into myofibroblasts, involving the process of tissue damage repair and fibrosis. Whether there is oxidative stress injury or inflammatory injury, it is often through the activation of myofibroblasts that leads to pulmonary fibrosis. The Collagen I and α-SMA expression levels are two important indicators of the fibrosis process. As an essential component of the ECM, high levels of Collagen I expression often indicate the onset of fibrosis. In 2021, Li et al. [32] obtained a 20-amino-acid peptide from PC (eicosapeptide) and investigated its anti-fibrotic effect on lung fibrosis. Their results demonstrate that eicosapeptide could dramatically depress the increase of Collagen I in HFL-1 cells induced by TGF-β1 and played an anti-fibrotic role. α-SMA is an important indicator of myofibroblast activation, and the increase in its expression signifies the activation of myofibroblasts and the development of pulmonary fibrosis [33]. Therefore, we investigated the inhibitory activity of phycocyanin peptides on the expression of α-SMA in HFL-1 cells as a complement to their anti-pulmonary fibrosis activity.

**Figure 8:**
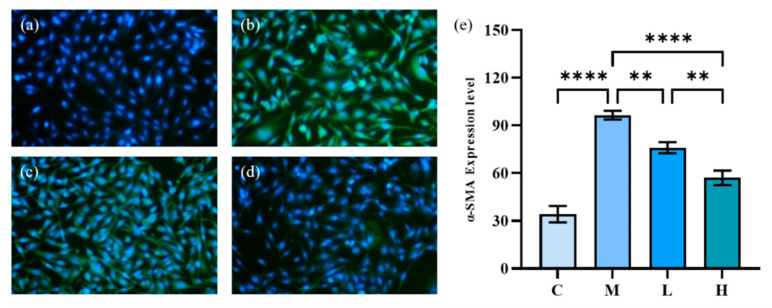
The effect of phycocyanin peptides on the expression of α-SMA in HFL-1 cells induced by TGF-β1: (**a**) the control; (**b**) the model; (**c**) low-dose at 10 μg/mL; (**d**) high-dose at 30 μg/mL (**e**) α-SMA expression level. C: the control group; M: the model group; L: the low-dose group (10 μg/mL); H: the high dose group (30 μg/mL). *n* = 3, mean ± SD. ** *p* < 0.01; **** *p* < 0.0001.

The results of immunofluorescence staining of α-SMA induced by TGF-β1 are shown in Figure 8, in which the green fluorescent part indicates the expression of α-SMA. The model group expressed a large amount of α-SMA after 72 h of TGF-β1 induction, and the cells were gradually transformed from fibroblasts to myofibroblasts. The green fluorescence of HFL-1 cells treated with phycocyanin peptides was reduced remarkably. At low doses, phycocyanin peptides could reduce the expression of α-SMA, while at high concentrations of phycocyanin peptides, α-SMA expression only appeared slightly, and returned to a normal level overall.

### 2.5. Covalent Docking

**Figure 9:**
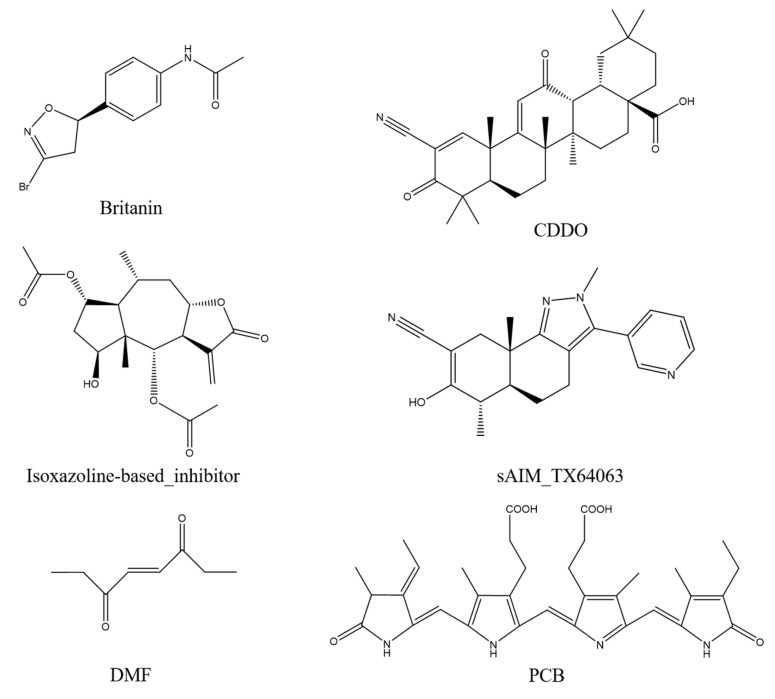
Keap1 small molecule inhibitor.

Britanin, CDDO, isoxazoline-based_inhibitor, DMF, and sAIM_TX64063 are all known small molecule inhibitors of the Keap1 BTB domain. Although the mechanisms of action of these small-molecule inhibitors vary, they can be grouped largely into three categories: (1) isoxazoline-based_inhibitor and DMF using α, β-unsaturated carbonyl groups as electrophilic receptors, (2) triterpenoids (CDDO and sAIM_Tx64063) taking nitrile group as electrophilic receptor, and (3) Britanin that uses a halogen as a reactive group and can suppress Keap1 activity by creating a covalent bond with the sulfur atom in Cys151 of the Keap1 BTB domain (Figure 9). PCB has α, β-unsaturated carbonyl groups, so we thought that PCB might also be able to inhibit the activity of Keap1 through the Michael addition reaction. DMF is considered to protect mouse neurons and astrocytes from oxidative stress; this process is thought to occur as DMF interacts with the Keap1 BTB domain to activate Nrf2 transcription, which in turn mediates the cellular antioxidant activity process [34]. Britanin is an herbal derivative that is thought to be capable of reversing hypoxia-glucose deprivation by inhibiting Keap1 [35]. As a triterpenoid, CDDO is thought to be a good inhibitor of various inflammatory diseases such as emphysema and COPD [36]. Isoxazoline-based–inhibitor is isoxazolinyl electrophile capable of reacting with cysteine residues, which has been used in the design of different covalent inhibitors [37]. Similar in structure to CDDO, sAIM_TX64063 is also a triterpenoid and has been demonstrated having an excellent activation effect on Nrf2, and this activity is also associated with the inhibition of Keap1 [38]. Therefore, these five small molecule compounds and PCB were selected for covalent docking simulations, and the possible anti-inflammatory and anti-fibrotic mechanisms of PCB were discussed by comparing the results after covalent docking.

**Figure 10:**
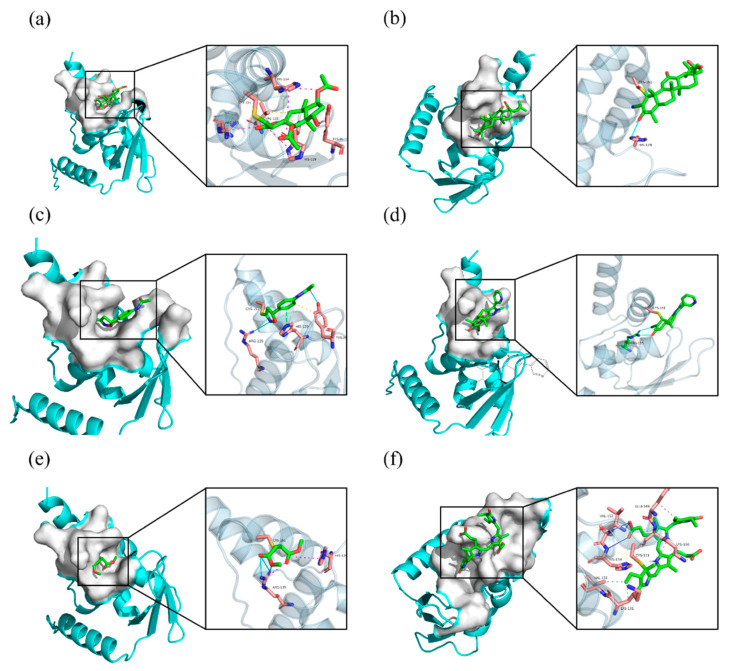
Small-molecule inhibitors covalent docking to the Keap1 BTB domain: (**a**) Britanin; (**b**) CDDO; (**c**) Isoxazoline-based–inhibitor; (**d**) sAIM_TX64063; (**e**) DMF; (**f**) PCB. The yellow dashed lines are hydrophobic interactions, the magenta dashed lines are salt bridges, the cyan solid lines are hydrogen bonds, and the green dashed lines are π-stacking.

Affinity (kcal/mol) shows the binding energy during covalent docking, and the lower the energy, the more effective the binding. It can be seen from the results that among the six small molecule compounds, PCB and Keap1 have the lowest binding energy of −3.8 kcal/mol, so the binding effect is the best, followed by Britanin with an affinity of −3.4 kcal/mol. CDDO and DMF are relatively poor, with an affinity of only −1.9 and −1.5 kcal/mol. The specific docking results are outlined in Table 3. The covalent docking map (Figure 10) also corroborates the results in Table 3. All of the six compounds were able to generate thioether bond with the Cys151 residue in the BTB domain of Keap1 to form a covalent bond. Among them, the steric hindrance between the conformations of PCB and Britanin and Keap1 is the smallest, so the relative affinity is low. However, CDDO and DMF interfere greatly with Keap1, resulting in high binding energy.

By comparing the covalent docking results of five known Keap1 inhibitors with PCB, it is not difficult to see that Britanin and PCB show more interactions with the amino acid residues of Keap1, so their binding energy is lower. As shown in Figure 10, Britanin can hydrophobically interact with TRY85, HIS129, VAL132, and HIS154 of Keap1, and form salt bridges with HIS129, ARG135, and HIS154. PCB can form six hydrophobic interactions with LYS131, VAL132, GLU149, and LYS150, generate salt bridges with LYS150 and HIS154, and form three hydrogen bonds with VAL152, LEU153, and HIS154. The isoxazoline-based–inhibitor can form two hydrogen bonds with TRY85 and ARG135 and generate π-stacking with the imidazole group of HIS129. The abundant interaction force reduces the binding energy of Britanin, PCB, and the isoxazoline-based–inhibitor when they are covalently bound to the receptor, so it is more favorable for their binding to Keap1. In contrast, CDDO forms only one hydrogen bond with HIS129, sAIM_TX64063 forms two hydrogen bonds with ARG135, and DMF forms salt bridges and hydrogen bonds with HIS129 and ARG135. Furthermore, the structure of CDDO would produce steric hindrance with the Keap1 protein, which enhances its binding energy. In summary, through the simulation of the docking of PCB and Keap1 molecules, it can be confirmed that PCB can activate Nrf2 by covalently binding to Keap1.

## 3. Discussion

Microalgae have been widely used for food and traditional medicine in the history of China. In 284–364 AD, Chinese alchemist Ge Hong discovered a filamentous blue algae and named it Gexianmi (*Nostoc sphaeroides*). Gexianmi is rich in nutrients, and can regulate immunity with anti-inflammatory and anti-tumor effects [39]. As a kind of microalgae, *Spirulina* has similar physiological activities as *Nostoc sphaeroides*, and the isolated PC from *Spirulina* is good for having antioxidant and anti-inflammatory activities.

As PCB in PC is sensitive to temperature, pH, and other ambient conditions, its color tends to change during the enzymatic hydrolysis process. To reduce the PCB loss of phycocyanin peptide as much as possible, we hydrolyzed PC with complex protease MC101 and controlled the reaction conditions to obtain pure blue phycocyanin peptide, and the yield was high, up to 96%. Compared with the commonly used phycocyanin peptides after enzymatic hydrolysis in the market, it was found that the colors of other enzymatic peptides mostly show purple brown and more blue-loss, while the phycocyanin peptides prepared with MC101 enzyme show bright blue-green, with good color retention, and the PCB contained still presented good activity.

The anti-inflammatory effect of PC was first proposed by Romay back in 1998, and they found that C-PC could inhibit liver microsomal lipid peroxidation [17]. Numerous experimental data have confirmed the usefulness of PC in various inflammation-induced diseases, such as acute lung injury [40], pulmonary fibrosis [18], allergic airway inflammation [41], alcoholic liver [42], liver injury [19], etc., showing certain degrees of relief and recovery effects.

**Figure 11:**
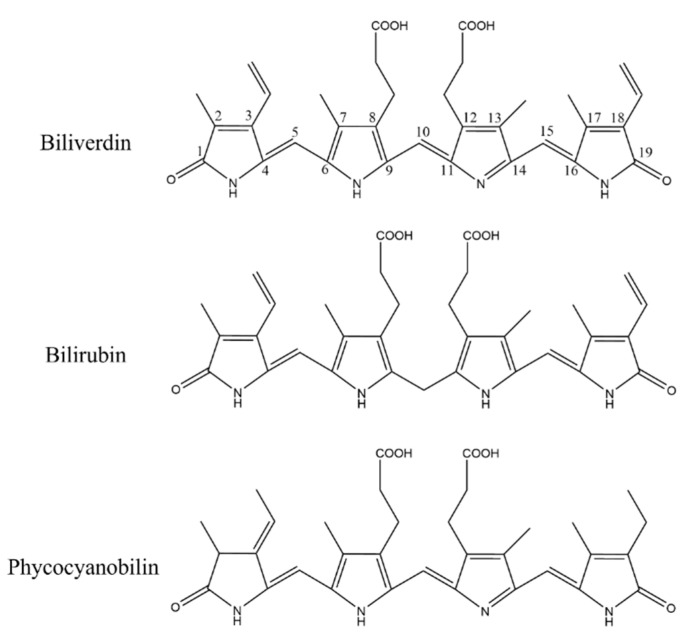
Structure comparison of biliverdin, bilirubin, and phycocyanobilin.

As a pigment protein from algae, PC contains a natural blue pigment called PCB. PCB is composed of four pyrrole rings connected by methylene pairs. It is very close to biliverdin in structure, with only the side chain substituents being slightly different, and its principal functional groups are consistent. In algae, the synthesis of PCB is through the ferrous heme metabolic pathway. Heme is oxidized to biliverdin IXα under the action of heme oxygenase (HO). It is different from biliverdin IXα in animals in that heme is reduced to bilirubin by biliverdin reductase. Biliverdin IXα in algae can be catalyzed with PCB reductase to form PCB [43], and then further connected with apoprotein to form PC. The structure of PCB is overall the same as that of bilirubin, except for slight alterations in the substituents of C3, C10, and C18; however, these differences did not affect the α, β-unsaturated carbonyl groups in the PCB (Figure 11).

HO-1 possess antioxidant and anti-inflammatory properties to modulate a variety of immune cells [20]. If the anti-inflammatory activity of HO-1 could be used and its expression be induced, it would provide us new ideas for the development of anti-inflammatory drugs. A traditional HO-1 inducer is metalloporphyrin, and it can significantly up-regulate the expression of HO-1; however, its toxicity cannot be ignored as it greatly limits its clinical application [20]. The reaction products of HO-1 can lead to the induction of HO-1 expression. Nam et al. [21] showed that bilirubin can induce the expression of Nrf2 and further induce the export of HO-1 located downstream of Nrf2 by binding to Keap1 through an electrophilic reaction with the α, β-unsaturated carbonyl group in the structure, which inhibits the binding of Keap1 and Nrf2. Similar to metalloporphyrin, bilirubin has a series of issues for clinical application. First, bilirubin can form intramolecular hydrogen bonds, resulting in the decrease in water solubility and becoming fat-soluble, which is not conducive to the dissolution when used as a drug. In addition, taking large amounts of bilirubin would increase the concentration of bilirubin in the body, causing hyperbilirubinemia (jaundice) and adversely affecting liver function [44].

**Figure 12:**
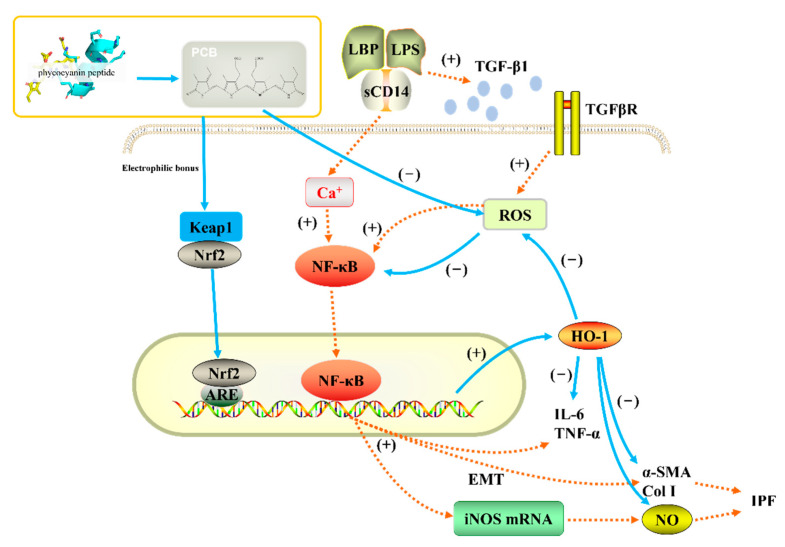
Anti-inflammatory pathway of phycocyanin peptide. The orange dotted line is the process of pulmonary fibrosis, and the blue solid line is the anti-pulmonary fibrosis mechanism of phycocyanin peptide.

Based on published scientific research, we believe that the antioxidant, anti-inflammatory, and anti-pulmonary fibrosis activities of phycocyanin peptides are related to the structure of the PCB bound in the phycocyanin peptide. As shown in Figure 11, PCB and bilirubin are only slightly different on the substituents of C3, C10, and C18. Therefore, PCB may have a similar effect on bilirubin *in vivo*, and the phycocyanin peptide could be quickly absorbed in the gastrointestinal tract because of its small molecular weight, after entering the body. The PCB bound in the phycocyanin peptide has a linear tetrapyrrole structure. They are polypeptides, and the resulting steric hindrance prevents the formation of internal hydrogen bonds between the propionic acid group and carbonyl group, which could increase the water solubility of PCB. After being absorbed by cells, the α, β-unsaturated carbonyl group on PCB act as electrophiles to bind the thiol of the cysteine residue in Keap1, release and activate Nrf2, transfer it to the nucleus, bind with ARE, and further activate the HO-1 gene. The activated HO-1 gene expresses HO-1, regulates immune cells [45], and inhibits the expression of inflammatory pathway NF-κB, which is under a direct anti-inflammatory effect (Figure 12). In addition, elevated HO-1 has antioxidant effects to eliminate ROS together with a direct antioxidant effect of the phycocyanin peptide to reduce oxidative stress damage in the tissue.

LPS can lead to the secretion of TGF-β1 by macrophages, and elevated TGF-β1 increases the level of ROS in cells [46] and induces lung fibroblasts to secrete ECMs, such as collagen, α-SMA, etc.; the cells develop to the state of myofibroblasts, and the tissues undergo fibrosis reactions. High levels of ROS activate the NF-κB pathway, mediate the secretion of cytokines, and aggravate inflammation and fibrosis among tissues [47]. HO-1 activated by PCB can directly eliminate ROS in the body, block the NF-κB pathway, and indirectly reduce tissue inflammation. A previous study of ours on the expression of related genes after phycocyanin peptide treatment shows that PC gavage of mouse could significantly increase the export of Nrf2 and the downstream gene HO-1, thereby reducing liver damage caused by radiation [19]. Meanwhile, Strasky et al. [48] showed that PCB could suppress the development of atherosclerosis by inducing the HO-1 expression, which supports the above assumptions to a certain extent.

## 4. Materials and Methods

### 4.1. Reagents

Phycocyanin was purchased from Zhejiang Binmei Biotechnology Co., Ltd., China. The compound protease MC101 was purchased from Yantai Maitel Biotechnology Co., Ltd., China (200,000 μ/g). RAW264.7 cells, CCK-8, and ELisa kits were purchased from Beijing Hua er bo si biology technology Co., Ltd., China. The Griess kits were purchased from Biyotime Biotechnology Co., Ltd., China. Immunofluorescence and the Western Blot antibodies were purchased from Beijing Hua er bo si biology technology Co., Ltd., and other reagents were of analytical grade.

### 4.2. Enzymatic Hydrolysis of Phycocyanin

Phycocyanin was hydrolyzed with compound protease MC101, and the enzyme addition was 5%. Other conditions were temperature 57 °C, pH natural, the ratio of material to liquid 1:80, and the reaction time 4 h. After digestion, the protease was removed by heating and centrifugation. The supernatant was suction-filtered with white diatomite, and the filtrate was vacuum freeze-dried.

### 4.3. Absorption Spectrum and Molecular Weight Distribution Measurement

Ultraviolet-visible spectrophotometer was used to measure the absorption spectrum of the phycocyanin peptide at 200–800 nm, and the distribution of the molecular weight of the phycocyanin peptide was determined using HPLC. The data were processed using GPC software. We used Agilent 1290 infinity II/6545 QTOF HPLC-MS to determine the fragments containing PCB in phycocyanin peptides and used Bioconfirm 8 to process the data.

### 4.4. Antioxidant Activity of Phycocyanin Peptide

#### 4.4.1. Superoxide Anion Clearance

As per Zhang and Meng et al. [49,50] with modification, phycocyanin peptide was configured in different concentrations with H_2_O. Tris-HCl of 0.05 M and pH 8.2, and 15 mM pyrogallic acid were kept in a water bath at 25 °C, and then 3.7 mL Tris-HCl, 1 mL phycocyanin peptide solution, and 0.3 mL pyrogallic acid were added. We shook the solution well, placed it into the spectrophotometer immediately, and measured the absorbance value every 30 s at a wavelength of 420 nm for a total of 4 min. We plotted the curve to find the slope *A_i_*.

(1) $$Clearance\;rate\;I\;\left(\%\right)=\frac{A_{c}-A_{i}}{A_{c}}\times 100\%$$

where *A_i_*—absorbance of reaction group; and *A*_c_—the absorbance of control group with deionized water instead of the phycocyanin peptide.

#### 4.4.2. ABTS^+^ Clearance

Refer to the experimental program of Mei et al. with modification [51].

Mix 38.40 mg ABTS with 6.623 mg K_2_S_2_O_8_, dissolve mixture with H_2_O and dilute to 10 mL, allow to react at room temperature in darkness for 12–16 h, dilute with 0.2 M, pH 7.4 PBS buffer to the absorbance of 0.70 at 734 nm ± 0.02 or so. We took 50 μL phycocyanin peptide solution and 150 μL ABTS^+^ solution and reacted in the dark for 6 min, then absorbance was measured at 734 nm.

#### 4.4.3. Reducing Power

Refer to the experimental program of Klomong et al. with modification [52].

Take 1 mL phycocyanin peptide, add 2.5 mL for each of 1% potassium ferricyanide solution and 0.2 M, pH 7.4 PBS buffer, water bath at 50 °C for 20 min, then add 2.5 mL 10% C_2_HCl_3_O_2_, mix well after centrifugation at 3000 rpm for 10 min. Take 2.5 mL of the supernatant, add 2.5 mL H_2_O and 0.5 mL 0.1% FeCl_3_ solution, stand still for 10 min, and detect the absorbance A_700_ at 700 nm and the value of A_700_ was used to reflect the magnitude of the reducing power.

### 4.5. Anti-Inflammatory Activity of Phycocyanin Peptide

#### 4.5.1. Cell Viability

Refer to Gao et al. [53]. Use DMEM medium to dilute the phycocyanin peptide to 50, 100, 200 μg/mL; take RAW264.7 cells at logarithmic growth, adjust the cell concentration to about 5 × 10^4^ cells/mL, and inoculate them in several 96-well plates, with 100 μL in each well; continue to incubate for 24 h at 37 °C in 5% CO_2_. After 24 h, renew the medium containing each group of drugs and continue to culture for 24 h; after adding the drugs for 24 h, renew the medium, add 100 μL to each well containing 10 μL CCK-8.

Prepare a medium reaction solution containing no cells as the zero well, incubate it for an appropriate time and measure the absorbance at 450 nm.

#### 4.5.2. Cytokines

Add 50 μL of 100 ng/mL LPS solutions to the treatment group and LPS group, respectively, and add the same volume of DMEM culture medium to the control group [54,55,56]. In the treatment group, different concentrations of phycocyanin peptide solution were added. After 24 h of culture, take 100 μL of the supernatant, use the NO detection kit to determine the NO content, and use the Elisa detection kit to determine the TNF-α and IL-6.

### 4.6. Determination of Anti-Pulmonary Fibrosis Activity of Phycocyanin Peptide

#### 4.6.1. Observation of A549 Cell Morphology

Select A549 cells as the cell model, and the cell culture conditions and groupings are shown in Table 4.

#### 4.6.2. Immunofluorescence

The expression of A549 cells Collagen I and HFL-1 cells α-SMA was measured by immunofluorescence.

After 72 h of continuous culture, 4% paraformaldehyde fixed, 1% TritonX-100 for 5 min and 3% H_2_O_2_ for 10 min. Secondary antibodies incubate for 2 h. DAPI nuclei stain for 5–10 min and photographed with a fluorescence microscope.

Use ImageJ software to analyze the gray value of immunofluorescence image and quantify its fluorescence intensity by its gray value.

#### 4.6.3. Western Blot

After the cells adhered to the wall, they were digested with 0.25% trypsin, seeded on a 6-well culture plate, and administered after the density reached 60%. The cells were observed after 72 h, washed with PBS, and scraped off with a cell scraper by adding deionized water. Cells were lysed, pipetted back and forth to mix, centrifuged to take supernatant, added with 5×loading buffer in proportion, and heated to 95 °C in a metal bath. Ten min later, the Western Blotting was performed to detect Nrf2, HO-1, NQO1, and EMT-related marker proteins.

### 4.7. Covalent Docking Analyze

The 3D structure of the Keap1 protein (BTB domain) was downloaded from https://www.rcsb.org, and the PDB ID is 7EXI. PyMol was used to remove water molecules. Five known small molecule inhibitors of Keap1 were selected: sAIM_TX64063 (PDB ID: 5DAF); 2-cyano-3,12-dioxooleana-1,9-dien-28-oic-acid (CDDO) (PDB ID: 4CXT); britanin (PDB ID: 5GIT); isoxazoline-based–inhibitor (PDB ID: 6FFM); dimethyl fumarate (DMF) (CAS NO. 624-49-7) and PCB (PubChem CID: 6438349) as ligands for covalent docking. Covalent docking of receptors and ligands was performed using the AutoDockFR program, and their conformations were optimized using a genetic algorithm based on the principle of minimum docking energy [57]. Interactions between receptors and ligands were analyzed using PLIP [58]. Results were visualized using PyMol.

### 4.8. Statistical Analysis

All experiments in this study were performed in triplicate, and results are shown as mean ± SD. Analyses were performed using SPSS Statistics 23 and graphs were constructed using GraphPad Prism 9.0. Data from two or more groups were analyzed using one-way ANOVA (*p* < 0.05 was deemed statistically significant).

## 5. Conclusions

The emergence of phycocyanin peptides has solved modern issues of HO-1 inducers and has the advantages of lower cytotoxicity and better water solubility. PCB in PC is similar in structure to bilirubin, so its related antioxidant, anti-inflammatory, and anti-pulmonary fibrosis activities were substantiated. Being bound to Keap1, PCBs can release Nrf2 and further induce HO-1 expression. Therefore, phycocyanin peptides can be used as a new HO-1 inducer to develop different types of anti-inflammatory and anti-pulmonary fibrosis drugs. However, the absorption and metabolism of PC, especially PCB, need to be further clarified. Future studies on the activity and bioavailability of PC will contribute to the industrial development of PC and phycocyanin peptides.

## Figures and Tables

**Table 1:** Molecular weight distribution of phycocyanin peptide.

Indicators	>10,000	10,000–5000	5000–3000	3000–2000	2000–1000	1000–500	500–180	<180
Peak area percentage (%, λ220 nm)	0.72	0.39	0.58	1.81	6.83	20.56	50.14	18.96
Number average molecular weight	13,929	7536	3476	2444	1267	639	253	–
Weight average molecular weight	14,429	7826	3539	2477	1311	661	271	–
total weight average molecular weight (Mw)	583							

–: no data.

**Table 2:** The fragments of phycocyanin peptide with PCB.

Seq Loc	Tgt Seq Mass	Sequence	Missed	Pred Mods
α(78-86)	1646.8351	QRGKDKCAR	8	PCB-α84
β(80-82)	849.3731	AAC	2	PCB-β82
β(81-84)	1047.5212	ACLR	3	PCB-β82
β(80-84)	1118.5583	AACLR	4	PCB-β82
β(81-86)	1293.5886	ACLRDM	5	PCB-β82
β(80-86)	1364.6257	AACLRDM	6	PCB-β82
β(152-155)	980.395	DCSA	3	PCB-β153
β(152-156)	1093.479	DCSAL	4	PCB-β153
β(148-155)	1348.6009	ITPGDCSA	7	PCB-β153
β(148-156)	1461.685	ITPGDCSAL	8	PCB-β153
β(147-155)	1405.6224	GITPGDCSA	8	PCB-β153

**Table 3:** Covalent docking results.

Mode	Affinity (kcal/mol)	Clust. RMSD	Ref. RMSD	Clust. Size	RMSD Stdv	Energy Stdv	Best Run
Britanin	−3.4	0.0	−1.0	7	0.4	0.2	003
CDDO	−1.9	0.0	−1.0	8	0.0	0.0	007
Isoxazoline-based–inhibitor	−3.1	0.0	−1.0	7	0.5	0.1	004
DMF	−1.5	0.0	−1.0	8	0.4	0.1	002
sAIM_TX64063	−2.1	0.0	−1.0	8	0.2	0.1	001
PCB	−3.8	0.0	−1.0	1	NA	NA	002

**Table 4:** Cell culture conditions and grouping.

	Control	Model	Low Dose	High Dose
Medium	1640	1640	1640	1640
FBS	10%	10%	10%	10%
Temperature	37 °C	37 °C	37 °C	37 °C
CO_2_	5%	5%	5%	5%
TGF-β1	–	10 ng/mL	10 ng/mL	10 ng/mL
Phycocyanin peptide	–	–	10 µg/mL	30 µg/mL
Sample size	100 µL	100 µL	100 µL	100 µL
Time	72 h	72 h	72 h	72 h

–: no data.

## Supplementary Materials

The following supporting information can be downloaded at: https://www.mdpi.com/article/10.3390/md20110696/s1, Table S1: Results of phycocyanin peptide HPLC-MS.
